# Distribution of Orchids with Different Rooting Systems in the Czech Republic

**DOI:** 10.3390/plants10040632

**Published:** 2021-03-26

**Authors:** Zuzana Štípková, Spyros Tsiftsis, Pavel Kindlmann

**Affiliations:** 1 Global Change Research Institute, Academy of Sciences of the Czech Republic, Bělidla 986/4a, 60300 Brno, Czech Republic; pavel.kindlmann@centrum.cz; 2 Faculty of Science, Institute for Environmental Studies, Charles University, Benátská 2, 12801 Prague, Czech Republic; 3 Department of Forest and Natural Environment Sciences, International Hellenic University, GR-66100 Drama, Greece; stsiftsis@for.ihu.gr

**Keywords:** diversity, altitude, orchid distribution, phytogeographical area, mean species specialization index

## Abstract

Understanding diversity patterns along altitudinal gradients and the effect of global change on abundance, distribution patterns and species survival are of the most discussed topics in biodiversity research. Here, we determined the associations of orchid species richness and the degree of their specialization to specific environmental conditions (expressed by species specialization index) with altitude in six floristic areas in the Czech Republic. We distinguished three basic trends in these relationships: linear, parabolic and cubic. We then determined whether these trends differ between three orchid groups classified by their rooting systems: rhizomatous, intermediate and tuberous. We used distributional data on 69 species and subspecies of terrestrial orchids recorded in the Czech Republic and interpolated them at 100-m intervals along an altitudinal gradient in each floristic area. The trends in both species richness and mean species specialization index differed between the six floristic areas within each of the three orchid groups studied. These patterns are probably strongly influenced by the orography of the country and the distribution of different habitats in the six floristic areas in the Czech Republic. We also found that the most widely distributed orchid group in the Czech Republic are the rhizomatous orchids, followed by intermediate and tuberous ones.

## 1. Introduction

Orchids are disappearing worldwide, mostly due to habitat loss, but other factors like climate change are likely to increase in importance during the 21st century [1,2]. Thus, one of the most worrying issues is that we still do not know the optimal abiotic and biotic requirements for population persistence of many of the ca. 30,000 species of orchids [3]. There are only a few studies in the Czech Republic dealing with the factors that determine orchid presence/absence and distribution in space, and most of them include only one or a few species and/or a limited part of the distribution of the species studied (e.g., [4,5]). We still lack critical information necessary for the conservation of Orchidaceae, especially those that are threatened or endangered.

Understanding the abundance and distribution patterns of species at large spatial scales is one of the key goals of biogeography and macroecology [6,7,8], but effective conservation requires knowledge of species at small spatial scales [9,10]. Thus, the knowledge of orchid ecology including ecological gradients that influence the patterns in orchid abundance, distribution, richness and composition is essential for planning and applying conservation strategies and actions [9,10], as lack of such knowledge negatively affects our ability to identify sites that are worth protecting.

There are two crucially important values when orchid conservation and survival under climate change is considered: number of species per unit area, and the degree to which an orchid species is specialized to specific environmental conditions. The former clearly determines the conservation value of the area, while the latter tells us how much a species may be endangered by changes of environmental conditions, e.g., by climate change.

The need for taking effective conservation measures is urgently required for areas and countries that have been affected by human activities in the past decades, and thus have lost a part of their biodiversity or their species distributions have been largely diminished [11,12]. This is especially true in the case of Central European countries, which have been intensively affected by land use change or agricultural intensification [11]. Among these countries, the Czech Republic was strongly affected by such changes over the last few decades. As a result, many orchids declined and can only be found at a small number of sites [12].

There are many publications on the distribution of orchids in the Czech Republic, which indicates that both professionals and the lay public are interested in orchids (e.g., [13,14,15,16,17,18,19]). However, there is only scattered information on the factors determining orchid distribution and species richness throughout the Czech Republic. Recently, Štípková et al. [20] determined the association between pollination mechanisms (presence/absence of nectar) and orchid species density and mean species specialization index along an altitudinal gradient in the six different phytogeographical regions in the Czech Republic.

**Figure 1:**
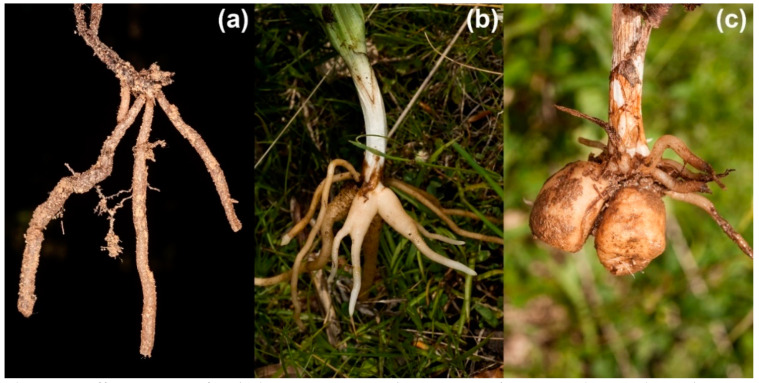
Different types of orchid root system: (**a**) rhizomatous, (**b**) intermediate and (**c**) tuberous.

In addition to their often specialized pollination strategies, orchids in temperate regions differ in their rooting systems, which are thought to represent particular strategies for underground storage of resources [21]. In this context, Tsiftsis et al. [8] report that the spatial patterns in the distribution of orchids in Greece are associated with their rooting systems, latitude, altitude and climate. They categorized orchids based on the morphology of their rooting system: (i) rhizomatous orchids (considered to be the most primitive), (ii) “intermediate orchids” (in evolutionary history intermediate between rhizomatous and tuberous orchids) and (iii) tuberous orchids (the most derived)—see Dressler [22], Averyanov [23] and Tatarenko [24] for reference. The typical shapes of terrestrial orchid roots in the three groups are depicted in Figure 1.

Tsiftsis et al. [8] report that species richness of orchids with these three rooting systems is significantly associated with altitude, whereas the degree to which an orchid species is specialized to specific environmental conditions (e.g., their mean species specialization index that will be described later here) are largely associated with their evolutionary history, represented by their rooting system.

However, these associations have not been studied in central Europe, where the climate is more continental and less variable than in countries in the south of Europe (e.g., Greece). The expectation is that the more continental climate in Central Europe would affect the distribution of species in a more uniform way. However, there are six different phytogeographical regions in the Czech Republic. They differ in altitude and consequently in their climatic conditions, but also in the spatial distribution of different habitats and their geological substrates [25], as well as in the intensity of human activities in the past. Thus, it is expected that these differences may also affect the distribution of orchids. Therefore, it is very important to analyze each of the phytogeographical regions separately, instead of all of them together.

To fill this gap in our knowledge, here we explore the associations of orchid species richness (adjusted for area considered) and the degree to which an orchid species is specialized to specific environmental conditions (measured as species specialization index—see its definition below) with altitude in the Czech Republic. For the reasons described above, we distinguish six phytogeographical regions and perform the analyses for each of them separately. We distinguish three basic trends in these relationships: linear, parabolic or cubic, and we then look at whether these trends differ between the three rooting systems studied here.

## 2. Results

**Figure 2:**
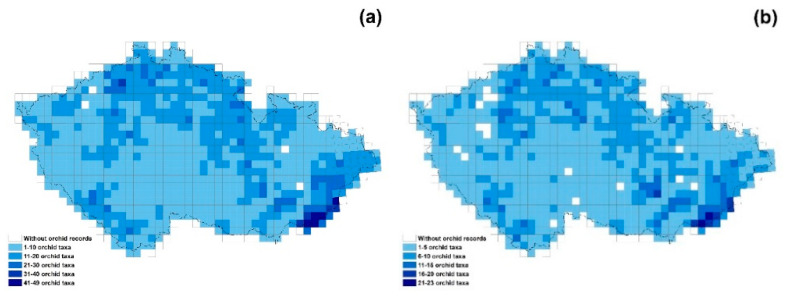

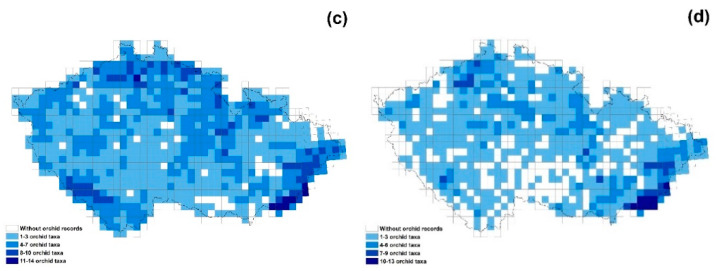
Maps of the Czech Republic showing the distributions of: (**a**) total number of orchid taxa, (**b**) rhizomatous, (**c**) intermediate and (**d**) tuberous orchid taxa.

Orchids were recorded in 858 (93.7%) of the 916 grid cells (Figure 2). The most widely distributed group of orchids is the rhizomatous species (present in 832 grid cells; 90.8% of the total area), followed by intermediate (distributed in 809 grid cells; 88.3% of the total area) and tuberous orchids (recorded in 616 grid cells; 67.2% of the total area). The most species-rich areas in the Czech Republic are in the south-eastern part of the country (Bílé Karpaty and Beskydy Nature Conservation Area), in the Šumava National Park in the south-western part, mountainous areas in the north on the borders with other countries (e.g., National Park of Krkonoše and České Švýcarsko, Jeseníky Nature Conservation Area) and some smaller inland areas (Figure 2a). Rhizomatous species (Figure 2b) occur almost across the whole of the Czech Republic, but the region with largest amount of species is found in Bílé Karpaty NCA, in the southeast part of the country. The most species-rich areas for intermediate orchids (Figure 2c) are also Bílé Karpaty and Beskydy NCA (south-eastern part of the country) and areas on the borders with other countries (in the north and southwest with Germany, in the north with Poland and in the south with Austria). For the tuberous species (Figure 2d), the highest species richness is also in the south-eastern part (mainly in the Bílé Karpaty NCA) and north-western part of the Czech Republic in the České Středohoří NCA. There are also small areas rich in tuberous species in Šumava NCA (south-western part of the country) and Vysočina region (southern territory of the central part of the Czech Republic).

**Figure 3:**
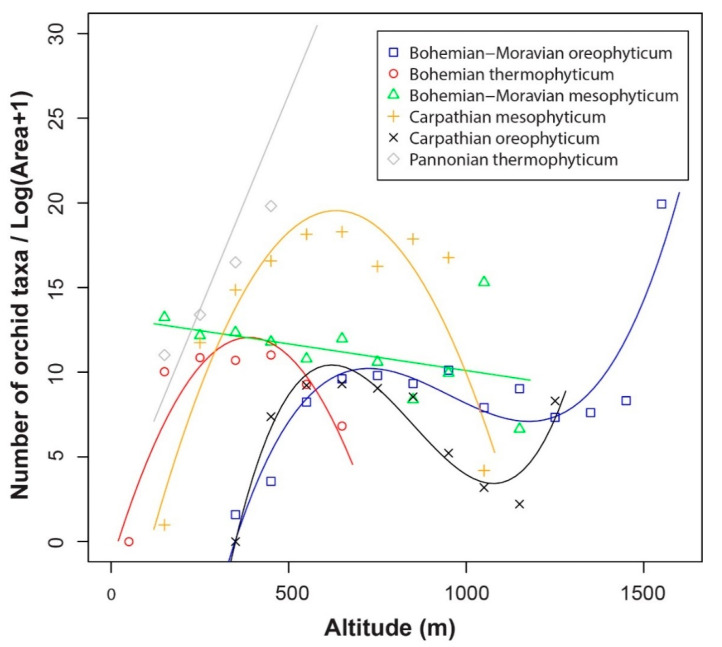
Orchid species density recorded along altitudinal gradients in each of the phytogeographical regions in the Czech Republic.

Figure 3 shows the associations between orchid density and altitude in each of the phytogeographical regions. In the Pannonian thermophyticum, orchid density increases with altitude. There are unimodal trends in Bohemian thermophyticum and Carpathian mesophyticum, where the species richness peaks at c. 400 m a.s.l. and 700 m a.s.l., respectively. Trends recorded in the Bohemian-Moravian and Carpathian oreophyticum are similar. For both areas, the highest species density was recorded at c. 600–700 m a.s.l. In the case of the Bohemian-Moravian oreophyticum species density decreased slightly up to c. 1200 m a.s.l., whereas in the Carpathian oreophyticum the numbers decreased rapidly up to c. 1100 m a.s.l. After these decreases in both areas, orchid density increased rapidly with increase in altitude. The predictive power of almost all regressions was good and the associations statistically significant, except in the case of the Bohemian-Moravian mesophyticum, where the results were not significant (Table 1).

**Figure 4:**
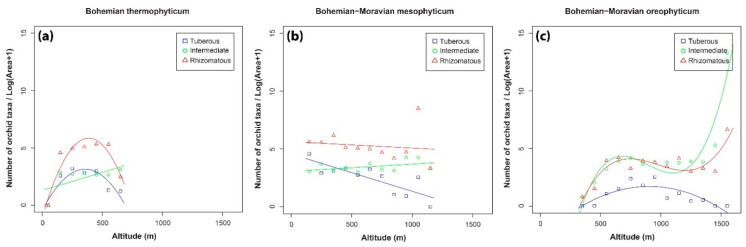

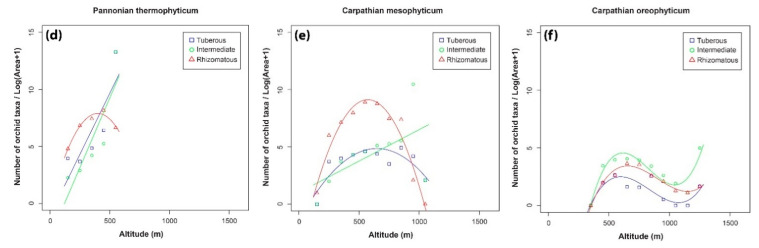
Density of tuberous, intermediate and rhizomatous orchid taxa recorded at different altitudes in the phytogeographical region of: (**a**) Bohemian thermophyticum, (**b**) Bohemian-Moravian mesophyticum, (**c**) Bohemian-Moravian oreophyticum, (**d**) Pannonian thermophyticum, (**e**) Carpathian mesophyticum and (**f**) Carpathian oreophyticum.

Orchid densities in terms of the number of tuberous, intermediate and rhizomatous orchid taxa in each phytogeographical region are displayed in Figure 4. Density of the rhizomatous and tuberous orchids is unimodally distributed with a peak around 400 m a.s.l. in the Bohemian thermophyticum followed by a decrease (Figure 4a). Density of intermediate orchid taxa increased with increasing altitude in this region. In the Bohemian-Moravian mesophyticum (Figure 4b), the total number of intermediate orchid taxa slightly increased with increase in altitude, whereas that of rhizomatous orchids decreased slightly, but both these trends were insignificant (Table 2). In contrast to the other two species groups, the density of the tuberous orchids in the Bohemian-Moravian mesophyticum decreased with increase in altitude. In biogeographical areas at high altitudes (Bohemian-Moravian oreophyticum), the trends for intermediate and rhizomatous orchids were almost the same (horizontally arranged S-shaped), whereas the number of tuberous orchids was much lower (Figure 4c). Densities of tuberous and intermediate orchids sharply increased with increase in altitude in the Pannonian thermophyticum (Figure 4d), whereas the trend for rhizomatous orchids was unimodal with the maximum at c. 400 m a.s.l. Intermediate orchid density increased with increase in altitude in the Carpathian mesophyticum (Figure 4e), but this relationship was not significant (Table 2). On the other hand, the trends for tuberous and rhizomatous orchids were unimodal with a peak at about 600 m a.s.l., with the rhizomatous orchids being the richest in terms of species. In the Carpathian oreophyticum, all groups (tuberous, intermediate and rhizomatous) showed similar trends (Figure 4f), and they only differed in the number of species of orchids recorded at each altitudinal interval. Specifically, for all groups, the number of orchid taxa increased with increase in altitude, peaking at about 600 m a.s.l., then decreased at different rates up to ca 1100 m a.s.l. before increasing again up to the highest altitudes.

**Figure 5:**
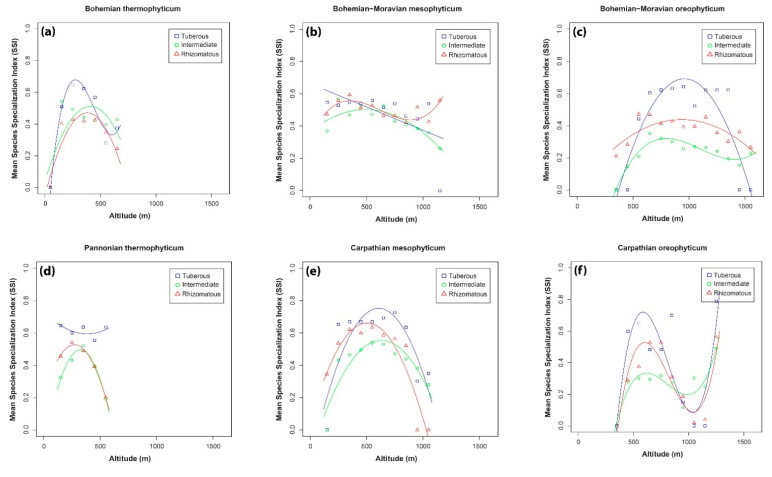
Relationships between mean species specialization index (*SSI*) and altitude recorded for the three orchid groups in the phytogeographical area of: (**a**) Bohemian thermophyticum, (**b**) Bohemian-Moravian mesophyticum, (**c**) Bohemian-Moravian oreophyticum, (**d**) Pannonian thermophyticum, (**e**) Carpathian mesophyticum and (**f**) Carpathian oreophyticum.

Figure 5 shows the relationships between mean species specialization index (represented by the values of mean species specialization index, *SSI*) and altitude of the three orchid groups in all phytogeographical areas in the Czech Republic. In general, intermediate and rhizomatous orchid groups show similar patterns in almost all phytogeographical regions, with hump-shaped or wave-shaped trends. In contrast, the pattern for tuberous orchids differed in all phytogeographical regions. In the Bohemian thermophyticum, a hump-shaped pattern was recorded for all orchid groups (Figure 5a), with tuberous orchids having a higher mean *SSI* value than the other two orchid groups, and the peaks occurred at lower altitudes (c. 250 m for tuberous orchids and c. 380 m for the rhizomatous and intermediate orchids). In the Bohemian-Moravian mesophyticum, there were no differences between the three groups (Figure 5b). In general, tuberous and intermediate orchids declined slightly, whereas the mean species specialization index of the rhizomatous orchids increased slightly at high altitudes. In the Bohemian-Moravian oreophyticum, tuberous orchids had much narrower niches than the other two orchid groups, but the humped-shaped curve is typical for species that occur at low and high altitudes and are characterized as extreme generalists (low *SSI* values) (Figure 5c). In contrast, the mean niche size of both rhizomatous and intermediate orchids remained almost stable along altitudinal gradients. In the Pannonian thermophyticum, the trends for rhizomatous and intermediate orchids were hump-shaped with maximums around 350 m a.s.l. (Figure 5d). Tuberous orchids were characterized by higher mean *SSI* values, but the results of the specific regression analysis were not significant (Table 3). The results recorded in the Carpathian mesophyticum were similar for all three orchid groups (Figure 5e). The curves of the *SSI* of the tuberous, intermediate and rhizomatous orchids had a hump-shaped distribution along altitudinal gradients with the highest number of specialists found between 500–700 m a.s.l. In the Carpathian oreophyticum, the shape of the three curves (corresponding to the three functional traits) was very similar and the differences were only in the max *SSI* values (Figure 5f). The most specialized species in all three groups were recorded at about 600 m a.s.l. and the incidence of specialization increased again at the highest altitudes in this phytogeographical area.

## 3. Discussion

### 3.1. Orchid Species Density along Altitudinal Gradients

The fact that species are not uniformly distributed across the globe stimulated Stevens [26] to write and publish his research on latitudinal Rapoport’s rule (LRR), which considers patterns of species distribution along latitudinal gradients, and a few years later he extended this hypothesis to include elevational gradient (elevational Rapoport’s rule, ERR [27]). Since then, a large number of studies have been published on different organisms (e.g., plants, animals) occurring in different areas of the world (e.g., [28,29,30,31]). It is worth mentioning that even for the same group of organisms, the explanations for the distributions differ considerably, so it is possible that the trends are associated with their functional groups (e.g., [8,20,32]).

The results presented support the existence of a hump-shaped curve; however, linear as well as cubic trends were also recorded. The highest species density was recorded between 300 and 900 m, which could be attributed to the fact that the distributions of many species of orchids overlap at these altitudes [32]. Naturally, phytogeographical regions in the Czech Republic do not have clear boundaries and there are many transitional zones between regions. Unlike the high number of species at mid altitudes, there are far fewer species at the highest altitudes, and they are mainly specialists that flourish in extreme conditions in mountains.

### 3.2. Patterns in the Distributions of the Three Orchid Groups

The distributions of each of the three orchid groups correspond to their specific ecological requirements, like altitude, type of bedrock or type of habitat, which confirm the patterns identified by Tsiftsis et al. [8] in southern Europe. In the Czech Republic, the most widely distributed are the rhizomatous orchids, closely followed by those with an intermediate root system. The rhizomatous orchids include genera like *Corallorhiza*, *Epipactis*, *Epipogium* and *Cephalanthera*, which mainly thrive in various kinds of forest habitats. This group has a wider distribution than the other two groups. It is likely that forest habitats, where rhizomatous orchids mostly occur, are more or less uniformly distributed throughout the Czech Republic. Like the rhizomatous orchids, intermediate orchids, such as *Dactylorhiza* spp., *Gymnadenia* spp. and *Platanthera* spp., occur in forests and open habitats. This means that many species occur almost everywhere in the Czech Republic. The least widely distributed group is the tuberous orchids. In general, tuberous orchids (species in the genera *Anacamptis*, *Ophrys* and *Orchis*) mainly grow in open habitats, such as grasslands or meadows. However, our results for this group differ from those reported for Southern Europe [8], where tuberous orchids are the most widely distributed group. Tuberous orchids evolved later than rhizomatous or intermediate orchids and are well adapted to the dry and hot climate around the Mediterranean, where their richness is really high [8,33]. As a result, they are better represented in southern countries, e.g., in the Mediterranean, compared to countries where the climate is continental, as in Central Europe. However, there are some tuberous orchids that are well adapted and occur in areas with a continental climate (e.g., *Orchis pallens*, *Traunsteinera globosa*, *Anacamptis morio*). Although such species can occur throughout the Czech Republic from a climatological point of view, they are restricted because they mostly prefer a calcareous substrate that does not occur everywhere. The large number of species of tuberous orchids recorded in the SE part of the country (Bílé Karpaty), however, could be attributed either to the presence of calcareous substrates, the extensive distribution of grassland communities or the higher temperatures there than in other areas in the Czech Republic. The significance of this area was identified in the 1990s and then subject to huge restoration program (in Bílé Karpaty NCA). About 500 hectares of arable land were converted back into dry or mesic grasslands, where tuberous orchids flourish, using a high-diversity regional seed mixture. Nearly all species sown successfully established and nearly half of unsown target species established spontaneously, including orchids. All of these grasslands are maintained by regular mowing, which favors grassland orchids by reducing the competition with the other herbaceous species of plants [34,35,36].

### 3.3. Orchid Species Richness in the Different Phytogeographical Areas

Previously, it was reported that the association of the composition of the orchid flora in the Czech Republic with altitude is much stronger than with biogeography [20], which differs from the results reported for other countries (e.g., Greece, Colombia) where different areas host different orchid taxa [8,37]. This may be attributed to the different distribution and height of mountains in the above-mentioned countries. In countries with high mountain ranges (e.g., Greece, Colombia), natural barriers can exist, which together with other factors can delimit the dispersal of orchid seeds from one place to another. In contrast, in the Czech Republic, the highest mountains are present mainly at the borders with other countries and are not so high (max 1600 m), so orchid seeds are able to be dispersed over long distances within the country.

In general, the trend in the density of species of orchids along altitudinal gradients is hump-shaped, but linear and cubic trends were also recorded. Density of species of orchids in the three groups differed in the six phytogeographical regions but the trends were similar in the Bohemian-Moravian mesophyticum and both oreophyticums, whereas in the other three regions the trends were different. These differences can be attributed to many aspects connected with the life history strategies of orchids. Orchids need vegetation in which they can grow and establish new populations from seeds. Thus, the spatial distribution of suitable vegetation has a big effect on the density of orchids in different regions [1,20,38,39,40]. In addition to vegetation, geological substrate plays an important role in determining the distribution of orchids [39]. Many tuberous orchids show a clear preference for areas with a calcareous substrate, whereas rhizomatous and intermediate orchids occur at sites with siliceous substrates. The same pattern is also recorded in Greece, where numbers of species of tuberous orchids increase with increase in the percentage of calcareous substrates within grid cells [8]. This may account for the difference in tuberous orchids recorded in the Bohemian part of the Czech Republic and in the Pannonian and Carpathian parts of these regions. Granite is frequent and there are only small calcareous areas, the preferred substrate for tuberous orchids, in the Bohemian part of the Czech Republic, whereas calcareous substrates are frequent in the Pannonian and Carpathian regions.

Mycorrhizal fungi and orchid pollinators are other important factors that indirectly affect orchid distribution. Distribution of mycorrhizal fungi in soil and of pollinators at different altitudes is expected to play a significant role. Orchids are strongly dependent on fungi for seedling germination, establishment and growth, so the distribution and diversity of orchids might depend on the associated fungal communities [41]. Although mycorrhizal associations are predominantly generalist, specialized mycorrhizal interactions have repeatedly evolved in orchids, indicating their potential role in limiting the geographical range of orchids [42]. It is mentioned by several authors that mycorrhizal fungi affect local plant distribution [43] and orchids are not an exception [44]. There is evidence that the abundance together with distribution of orchid mycorrhizal fungi are important drivers of orchid density [45]. However, more studies on orchid mycorrhizal fungi are needed for a better understanding of the factors shaping the distribution of mycorrhizal fungi along altitudinal gradients [46].

Distribution of pollinators differs with altitude, with high altitude areas being the poorest [47]. Such differences may play an important role in pollination success, with orchids at low altitudes producing more fruit (and seeds) than those at high altitudes. This is reported for Hungary by Gilián et al. [48], where the reproduction success of the two lowest mountain populations of *Cephalanthera rubra* was extremely high (83–85% of the flowers produced fruit), whereas populations at higher altitudes produce significantly fewer fruits (about 40% of the flowers produced fruit). Populations at high altitudes might be limited by pollinators as well as by climatic conditions (such as low temperature and irradiance). Thus, temperatures and subsequent climatic conditions in different phytogeographical regions also play a role in the density of the three orchid groups.

### 3.4. Relationship between Mean Species Specialization Index and Altitude

The general distribution of each of the three orchid groups is determined by the specific ecological requirements of all species in each group, the spatial distribution of different habitats and the presence of pollinators and mycorrhizal fungi [49,50]. We used the species specialization index (*SSI*). *SSI* was calculated using the climatic conditions in all the places in the Czech Republic where an orchid occurred. In general, the breadth of a species niche increases with increase in altitude [51]. However, Tsiftsis et al. [8] in their study on Greek orchids found that these trends are mostly associated with orchid life forms. Similar to Tsiftsis et al. [8], we also recorded different trends in the three species groups and six phytogeographical areas in the Czech Republic.

We assume that the differences in the trends in the phytogeographical regions might be based on the distribution patterns of orchids specific to a given area. In the current study, the majority of the specialized species of orchids were recorded at low to middle altitudes. However, most regression curves were typically hump-shaped, which means that species recorded at high and low altitudes are generalists, as they are recorded at sites with a wide range of environmental conditions. This is in accordance with our previous study in which different pollination strategies of orchids were considered [20]. However, there are some differences in the occurrence of specialist and generalist orchids. In the Bohemian-Moravian mesophyticum, the trend recorded for the intermediate group supports the previous statement, whereas the distribution patterns of rhizomatous and tuberous orchids differed slightly. The most specialized species of rhizomatous orchids (such as *Epipactis pontica*) were recorded also at the highest altitudes in this region, and in the case of tuberous orchids, the incidence of specialist species decreased with increase in altitude and is not hump-shaped. Another example of a difference are the trends for the species distributed in oreophyticums. In both areas, intermediate orchids were the least specialized and tuberous orchids the most specialized orchid group. This specific pattern may be attributed to the ecological requirements of these species. In both areas, there are tuberous orchids with narrow species specialization index (e.g., *Anacamptis pyramidalis*, *Ophrys insectifera* or *Neotinea tridentata*), which occur more frequently in southern Europe [8] and are rather rare at low and medium altitudes in both oreophyticums. In the case of Carpathian oreophyticum, the trends for all species groups only differ slightly. The most specialized species (the rhizomatous *Cephalanthera rubra*, intermediate *Pseudorchis albida* and tuberous *Anacamptis morio*) occur at low (ca. 600 m) and the highest altitudes in this phytogeographical region. At other altitudes, orchids are generalists, occurring in a wide range of environmental conditions. The narrower niches of tuberous orchids compared to rhizomatous and intermediate orchids could be due to the distribution of forest and grassland habitats at particular altitudes. In the Carpathian oreophyticum, there are more forests at low and high altitudes, which are replaced by grassland at middle altitudes, where tuberous orchids typically occur. These grasslands are currently maintained by traditional management, mainly pastoralism. At the highest altitudes, this area is covered by alpine vegetation that is unsuitable for orchids, with only specialist orchids occurring in these harsh conditions.

In general, the differences in the distribution of specialists and generalists within each group might be due to the very different distributions of open and forested habitats in the phytogeographical regions. In the Czech Republic, the middle and high altitudes are abundantly covered by forests [19], where most rhizomatous and several of the intermediate orchids typically occur. This is in accordance with our results: rhizomatous and intermediate orchids were more widely distributed in floristic areas at high altitudes (mesophyticums and orephyticums). However, natural non-forested areas are also present in the highest altitudinal zone in the Carpathians [19,52], where most specialist tuberous orchids occur. Open habitats are typically inhabited by tuberous and some of the intermediate orchids. Some species in these groups belong to the most endangered orchid taxa in the Czech Republic [53]. We fully agree with Jacquemyn et al. [49] that the extinction of orchids is significantly related to the survival of the habitats where these orchids occur.

Distribution of habitats is closely connected with the distribution of orchid pollinators. Some orchids, mainly those occurring in forests, rely on pollinator being present to produce seeds [54]. The abundance of pollinators is declining all over the world [55] and is influenced by climate (e.g., temperature and seasonality) in a given area, which in turn is strongly determined by altitude [47]. Another factor influencing species specialization index is orchid rarity. Previously, it was reported that a nectar reward affects rarity and the probability of extinction of orchids [20,49,56,57]. Similarly, in south-western Australia, sexually deceptive taxa are more often rare than food-rewarding taxa [58]. Conservation efforts will be most effective if they combine ex situ strategies at locations with high habitat conversion rates and reservation strategies in rarity and richness hotspots, particularly where they overlap [59]. Moreover, in the Australian hotspot, the Southwest Australian Floristic Region, the greatest number of rare taxa occurred in areas of high taxon richness and naturally fragmented edaphic environments [58].

Another important finding of the present study is the mismatch in the number of species and *SSI* values. Comparing Figure 4 with Figure 5 reveals that in most areas with high species richness (expressed in terms of orchid density) are associated with low values of the mean species specialization index, and vice versa. This could be attributed to the fact that in areas with a high number of species, most species or a considerable number of species are widely distributed with low *SSI* values, which results in low values for the mean *SSI*. In contrast, in areas where only a few species were recorded (e.g., tuberous orchids in the Bohemian-Moravian oreophyticum), most of these species are not widespread in the Czech Republic, and as a result their *SSI* values are high. It could be hypothesized that the ecological conditions in areas with just a few species are extreme (e.g., climatic, soil) and consequently only a small number of species can occur there and nowhere else.

## 4. Materials and Methods

### 4.1. Areas Studied

**Figure 6:**
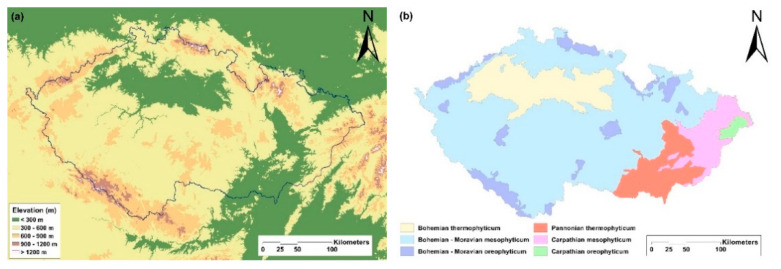
Maps of the Czech Republic showing: (**a**) the areas at different altitudes and (**b**) phytogeographical areas (taken and modified from Kaplan 2012).

The Czech Republic (N 48°33′–51°03′, E 12°05′–18°51′; area: 78 866 km^2^; altitude: 115–1602 m a.s.l.) is situated in the middle of Europe and its orchid flora is very well studied by many orchid enthusiasts and researchers. It is covered mainly by hillsides and highlands and the vast majority of the highest altitudes and mountains occur along the borders with other countries, especially in the north and south (Figure 6a). The average altitude of the country is 450 m a.s.l., which is slightly above the average for Europe (290 m a.s.l.). The climate in the Czech Republic is typically temperate with cold, cloudy and humid winters and hot summers, although there are some regional and local differences due to the relief that increases the topographic complexity. In view of the fact that the Czech Republic is a relatively small country, temperature and precipitation are more affected by local vertical heterogeneity and altitude than latitude.

Based on the phytogeographical division proposed by Kaplan [25], the Czech Republic is divided in three phytogeographical regions based on the dominant flora and vegetation that reflects specific regional geomorphological and climatic conditions—Thermophyticum, Mesophyticum and Oreophyticum. Thermophyticum includes warm areas with a thermophilous flora and vegetation that occur mainly in the lowlands. This region is characterized by the occurrence of basiphilous thermophilous oak and oak-hornbeam forests, dry scrubland and grassland (*Festuco-Brometea* class), whereas peat bogs and beech forests are nearly absent. The remnants of softwood floodplain forests, loess deposits, calcareous fens, as well as local saltmarshes and saline meadows, can be found there. The flora and vegetation in the Mesophyticum are typical of the Czech Republic and Central European temperate zone, and occur in the foothills or on lower slopes of the mountains (sub-mountain belt). The potential natural vegetation in this region consists mainly of various types of mesic beech or hornbeam forests, meadows and grassland, typically with *Arrhenatherum elatius*, *Molinia caerulea* and *Bromus erectus*, herbaceous forest edges and some specific communities, such as the vegetation growing on the exposed bottoms of fishponds. The last phytogeographical region, the Oreophyticum, is cold with a mountainous flora and vegetation, corresponding to the forests in the boreal zone, but also with smaller areas above the timberline similar to habitats in the arctic. Typical vegetation comprises mainly coniferous forests or mixed forests with a high abundance of conifers. Natural subalpine and alpine grassland (above timberline) occur only at the highest altitudes, where montane and sub-boreal species can be found [19,25]. Each of these regions is further divided into two provinces.

As a result, in the Czech Republic, there are the following six phytogeographical regions: (a) Bohemian thermophyticum, (b) Pannonian thermophyticum, (c) Bohemian-Moravian mesophyticum, (d) Carpathian mesophyticum, (e) Bohemian-Moravian oreophyticum and (f) Carpathian oreophyticum (Figure 6b). For descriptions of the individual phytogeographical areas, see Štípková et al. [20].

### 4.2. Dataset

The dataset of orchid records was based on the database of the Nature Conservation Agency of the Czech Republic. Classification and nomenclature of the taxa studied follows Danihelka et al. [17], apart from *Dactylorhiza fuchsii* subsp. *carpatica* (Batoušek & Kreutz) Kreutz, which was also included in the species list as it is now an accepted taxon [60].

We classified orchids into one of three root systems, namely rhizomatous, intermediate and tuberous, following the concept presented by Tsiftsis et al. [8]. Specifically, the genera *Cephalanthera*, *Corallorhiza*, *Cypripedium*, *Epipactis*, *Epipogium*, *Goodyera*, *Hammarbya*, *Limodorum*, *Liparis*, *Malaxis* and *Neottia* are classified as rhizomatous species, and the genera *Dactylorhiza*, *Gymnadenia*, *Platanthera* and *Pseudorchis* as intermediate. Those of the genera *Anacamptis*, *Herminium*, *Himantoglossum*, *Neotinea*, *Ophrys*, *Orchis*, *Spiranthes* and *Traunsteinera* are classified as tuberous orchids.

### 4.3. Statistical Analyses

First, the altitude of each orchid record was extracted from the altitudinal layer (30-sec resolution; approximately 1 km^2^), available through the WorldClim database [61]. Second, the altitudes in each phytogeographical area were divided into 100-m (e.g., 0–100 m, 101–200 m, etc.) vertical intervals, and third, the area (in km^2^) of each 100-m interval was calculated by counting the number of the 30-sec grid cells in the altitudinal layer with values in that interval. An orchid was considered as present in a 100-m interval only in the case where it was recorded at least once in a specific vertical interval.

In macroecological studies, species are considered that have continuous distributions [62]. However, here we did not follow this assumption, because—at a local scale—species may not be continuously distributed due to unsuitable ecological conditions, e.g., grassland species do not occur in forested areas and vice versa. After compiling the total matrix for all the orchid taxa recorded in the Czech Republic, a series of orchid matrices were generated based on the traits studied. Specifically, for each orchid category (rhizomatous, intermediate, tuberous), the number of orchid taxa occurring in each vertical interval was counted. In order to check for spatial differences within the Czech Republic, it was divided into the identified phytogeographical areas [25] and the process described above was repeated separately for each of these areas.

To adjust orchid species richness for area, we have calculated orchid density, D, at each altitudinal interval from the formula:

D = S/log(A + 1),(1)

where S is the number of orchid taxa recorded in each vertical interval and A is the area of each vertical interval.

To get a measure of species tolerance (often also called species niche breadth) for each species and floristic region, we used the Outlying Mean Index analysis described by Dolédec et al. [63]. Simply speaking, species tolerance measures the range of environmental conditions that the species can “tolerate” without going extinct; see Dolédec et al. [63] for details. For presenting the results, we used its complement: species specialization index (*SSI*):

*SSI* = 1 − T_i_/T_max_, (2)

where, T_i_ is the tolerance (%) of i_th_ species, and T_max_ is the maximum value recorded for a species tolerance (%). We have used *SSI*, because its values are between 0 and 1 (contrary to species tolerance) and it also has a more straightforward biological meaning: large *SSI* means that a species occurs in habitats with a limited range of conditions (specialist species), whereas low *SSI* means that a species occurs in habitats with widely varying environmental conditions (generalist species).

Outlying Mean Index analysis was used for each phytogeographical region by considering all the orchids occurring in each area, as well as the 19 bioclimatic variables and altitude of a specific area. After calculating the *SSI* values for each species in each area, we calculated the sum of the *SSI* values for each 100-m vertical interval on the basis of the species in each group recorded in the specific interval. As a final step, the mean *SSI* values were calculated by dividing the sum of the values by the number of orchids in each group recorded in each vertical interval.

In order to explore the associations of orchid density, D, and mean species specialization index, *SSI*, with altitude, we used regression. As we did not have any a priori hypothesis about the functions describing the shape of the dependences studied, polynomial regressions were used. We first used third-degree polynomials. If the cubic term was not significant, we used quadratic regression. If even the quadratic term was insignificant, we used linear regression [8,20].

All analyses were performed in R version 4.0.2 [64], whereas variable extraction was done using ArcGIS 10.1 [65]. Specifically, species tolerance was calculated using *ade4* package [66], whereas polynomial regressions were fit to the data using *stats* package, which is part of R software.

## 5. Conclusions

Knowledge of orchid ecology, including ecological gradients that influence patterns of orchid abundance, distribution, richness and composition, is essential for planning and applying conservation strategies and actions. Orchids are disappearing worldwide, including in the Czech Republic. The distributions of many species of Czech orchids have decreased markedly over time because of excessive use or alteration of their habitats mainly due to human activity.

In the current study, the most widely distributed orchid group was the rhizomatous orchids, closely followed by intermediate orchids. Orchids from both groups mainly occur in forested habitats, of which only a small part is protected. As in our previous study on orchid distribution patterns based on pollination systems, we emphasize the need to conserve forest habitats, which host many species of orchids, in addition to open habitats such as meadows.

Based on our results, we assume that the distribution of orchid taxa in each group strongly depends on the distribution of suitable habitats and types of bedrock, together with mycorrhizal fungi, at different altitudes.

A more complex assessment of the factors affecting the distribution of orchids locally and globally will depend on the results of further studies on the relationships between patterns in orchid distribution and that of mycorrhizal fungi.

## Figures and Tables

**Table 1:** Summary statistics of the polynomial regressions used for determining the relationship between the total number of orchid taxa and altitude present in each phytogeographical region.

Biogeographical Region	R^2^	*p* Value
Bohemian thermophyticum	0.75 (b)	*p* < 0.05
Bohemian-Moravian mesophyticum	0.11 (a)	ns
Bohemian-Moravian oreophyticum	0.80 (c)	*p* < 0.001
Pannonian thermophyticum	0.79 (a)	*p* < 0.05
Carpathian mesophyticum	0.81 (b)	*p* < 0.001
Carpathian oreophyticum	0.86 (c)	*p* < 0.001

(a): 1st order polynomial regression; (b): 2nd order polynomial regression; (c): 3rd order polynomial regression; ns: not significant.

**Table 2:** Summary statistics of the polynomial regressions used for determining the relationship between orchid diversity and altitude.

Root System	Biogeographical Region	R^2^	*p* Value
Tuberous	Bohemian thermophyticum	0.69(b)	*p* < 0.05
	Pannonian thermophyticum	0.63(a)	*p* < 0.05
	Bohemian-Moravian mesophyticum	0.64(a)	*p* < 0.01
	Carpathian mesophyticum	0.62(b)	*p* < 0.05
	Bohemian-Moravian oreophyticum	0.59(b)	*p* < 0.01
	Carpathian oreophyticum	0.55(c)	*p* < 0.05
Intermediate	Bohemian thermophyticum	0.31(a)	*p* < 0.05
	Pannonian thermophyticum	0.66(a)	*p* < 0.05
	Bohemian-Moravian mesophyticum	0.12(a)	ns
	Carpathian mesophyticum	0.27(a)	ns
	Bohemian-Moravian oreophyticum	0.83(c)	*p* < 0.001
	Carpathian oreophyticum	0.78(c)	*p* < 0.01
Rhizomatous	Bohemian thermophyticum	0.80(b)	*p* < 0.05
	Pannonian thermophyticum	0.95(b)	*p* < 0.05
	Bohemian-Moravian mesophyticum	0.09(a)	ns
	Carpathian mesophyticum	0.92(b)	*p* < 0.001
	Bohemian-Moravian oreophyticum	0.69(c)	*p* < 0.01
	Carpathian oreophyticum	0.93(b)	*p* < 0.001

(a): 1st order polynomial regression; (b): 2nd order polynomial regression; (c): 3rd order polynomial regression; ns: not significant.

**Table 3:** Summary statistics of the polynomial regressions used for determining the relationship between mean Species Specialization Index (*SSI*) and altitude.

Biogeographical Region	Root System	R^2^	*p* Value
Bohemian thermophyticum	Tuberous	0.91(c)	*p* < 0.01
	Intermediate	0.27(b)	ns
	Rhizomatous	0.73(b)	*p* < 0.05
Bohemian-Moravian mesophyticum	Tuberous	0.28(a)	*p* < 0.05
	Intermediate	0.69(b)	*p* < 0.01
	Rhizomatous	0.45(c)	*p* < 0.05
Bohemian-Moravian oreophyticum	Tuberous	0.77(b)	*p* < 0.001
	Intermediate	0.87(c)	*p* < 0.001
	Rhizomatous	0.51(b)	*p* < 0.01
Pannonian thermophyticum	Tuberous	0.38(b)	ns
	Intermediate	0.93(b)	*p* < 0.05
	Rhizomatous	0.98(b)	*p* < 0.01
Carpathian mesophyticum	Tuberous	0.63(b)	*p* < 0.01
	Intermediate	0.73(b)	*p* < 0.01
	Rhizomatous	0.84(b)	*p* < 0.001
Carpathian oreophyticum	Tuberous	0.54(c)	*p* < 0.05
	Intermediate	0.75(c)	*p* < 0.01
	Rhizomatous	0.74(c)	*p* < 0.01

(a): 1st order polynomial regression; (b): 2nd order polynomial regression; (c): 3rd order polynomial regression; ns: not significant.

## Data Availability

Restrictions apply to the availability of these data. Data were obtained from the Nature Conservation Agency of the Czech Republic and are available at https://portal.nature.cz/publik_syst/ctihtmlpage.php?what=3&nabidka=hlavni&variantaPrihlaseni=ISOP with the permission of the Nature Conservation Agency of the Czech Republic.
